# Early Identification of DLD in Paediatric Practice: A Pilot Validation of the CLAP Screening Tool in Italian Outpatient Settings

**DOI:** 10.1111/1460-6984.70282

**Published:** 2026-06-25

**Authors:** Andrea Ricotti, Danilo Dimitri, Federica Nebbia, Andrea Carpino, Lorena Charrier, Giulia De Cillis, Silvia Bonzo, Maria Actis, Elena Grosso, Emanuela Malorgio, Silvia Gambotto, Lucia Borsotti, Silvia Gallo, Fabiana Marnetto, Emilia Parodi

**Affiliations:** ^1^ Clinical Trial Unit Ordine Mauriziano Hospital Torino Italy; ^2^ Department of Clinical and Biological Sciences University of Torino Orbassano Italy; ^3^ Department of Psychology Salesian University Institute Torino Rebaudengo Torino Italy; ^4^ ASL CN1 Child Neuropsychiatry Cuneo‐Mondovì Cuneo Italy; ^5^ Easy‐Brain APS Torino Italy; ^6^ Pediatric Emergency Unit Regina Margherita Children's Hospital Torino Italy; ^7^ Department of Public Health and Pediatrics University of Torino Torino Italy; ^8^ Speech Therapy Degree Program University of Torino Torino Italy; ^9^ SICuPP (Società Italiana delle Cure Primarie Pediatriche) Torino Italy; ^10^ Pediatric and Neonatology Unit, Ordine Mauriziano Hospital Torino Italy

**Keywords:** developmental language disorder, early identification, item response theory, language development, paediatrician, screening, validation

## Abstract

**Background:**

Language development in early childhood varies considerably, making early detection of Developmental Language Disorders (DLDs) challenging despite their high prevalence and long‐term effects on learning and mental health. In Italy, no culturally adapted, easy‐to‐use screening tools are currently available in primary care. To address this gap, a screening tool was developed to support the early identification of children aged 24–72 months at risk of DLD and other clinically relevant language difficulties.

**Aims:**

To evaluate the psychometric properties and accuracy of the Comunicazione e Linguaggio in Ambulatorio Pediatrico (CLAP), a brief age‐specific screening tool designed for use in Italian paediatric outpatient settings.

**Methods and Procedures:**

In this pilot validation study, children were recruited by primary care paediatricians during routine well‐child visits and stratified into four age groups: 24–30, 36–42, 48–54, and 60–72 months. After administration of the CLAP screening tool, each child underwent a blinded speech‐language pathologist (SLP) assessment. Psychometric evaluation included internal consistency, item–total correlations, confirmatory factor analysis, and item response theory indices (discrimination and difficulty). Diagnostic accuracy was assessed using ROC curves, area under the curve (AUC), sensitivity, specificity, and optimal cut‐offs. Analyses were conducted separately for each age group.

**Outcomes and Results:**

Fifty children were enrolled in each age group; overall, 24% of the sample fell into the pathological subgroup after the blinded SLP assessment. Internal consistency was acceptable in the 24–30‐month (KR‐20 = 0.695) and 36–42‐month (KR‐20 = 0.777) groups, but lower in older children. Factor analyses supported a mainly unidimensional structure in the younger groups. Item response theory showed good discrimination and informativeness for several items. ROC analyses indicated excellent diagnostic accuracy in the 24–30‐month group (AUC = 0.93; sensitivity = 92%; specificity = 87%), fair accuracy in the 36–42‐ and 48–54‐month groups (AUC = 0.75 and 0.74), and poor performance in the 60–72‐month group (AUC = 0.46).

**Conclusion and Implications:**

The CLAP demonstrates promising psychometric properties and good‐to‐fair accuracy as a brief screening tool for identifying children aged 24–54 months at risk of clinically relevant language difficulties, including those who may need further assessment for DLD. Its age‐specific design, quick administration, and non‐invasive nature support its potential integration into routine primary care. For older children, an age‐specific revision or an alternative tool might be required. A larger validation study is currently in progress.

**WHAT THIS PAPER ADDS:**

*What is already known on this subject*

- Developmental Language Disorder (DLD) is common in early childhood; however, early identification remains difficult due to variable developmental pathways and the absence of validated screening tools in primary care. Currently, no brief, culturally adapted instrument is available for routine use in Italian paediatric settings.

*What does this study add to existing knowledge*

- This study demonstrates that the CLAP tool has promising psychometric properties, with good accuracy in the youngest age group and fair accuracy up to 54 months for identifying children at risk of clinically relevant language difficulties, including those who may later meet criteria for DLD. It provides the first evidence supporting an age‐specific, feasible screening option that can be integrated into Italian primary care, while also identifying areas requiring revision for older pre‐schoolers.

*What are the potential or actual clinical implications of this work?*

- CLAP can assist paediatricians in the early detection of clinically relevant language difficulties in children during routine well‐child visits. Its adoption could help standardize early language screening in Italy, leading to earlier referral for further diagnostic assessment and appropriate speech–language evaluation.

## 1 Introduction

Significant variability in language development trajectories is commonly observed in early childhood. Some children consistently show low language skills, while others outgrow early delays; some develop typically but later present with late‐emerging language disorders. This dynamic aspect of language acquisition complicates early detection of language disorders (Sax and Weston 2007; Paul et al. 2018; Gard et al. 1993). Language disorders that appear without other neurodevelopmental conditions, sensory deficits (e.g., hearing loss), or major cognitive delays are known as Developmental Language Disorders (DLDs). DLDs primarily affect a child's ability to understand and/or produce language, making them among the most common developmental conditions in preschool children, with prevalence estimates in high‐income countries ranging from 5% to 10% (Bishop et al. 2017). If left untreated, these disorders can significantly affect cognitive, academic, behavioural, and mental health outcomes throughout childhood and beyond (Snowling et al. 2016). Despite their high prevalence and long‐term effects, universal screening for language disorders is not routinely part of standard child health surveillance programs (Sim et al. 2019; Othman 2021). In practice, paediatricians often rely on developmental milestone charts as informal guides during well‐child visits for children aged 0 to 60 months (Misirliyan et al. 2023). Several international screening tools have been developed and may be suitable for use in primary care (Lavesson et al. 2018; Law and Harris 2000; Kas et al. 2022; Visser‐Bochane et al. 2021; Wallace et al. 2015). However, none are easily adaptable to Italian or directly compatible with the workflow of a typical paediatric outpatient clinic. Consequently, Italy currently lacks a standardized, user‐friendly, and easily integrable screening protocol for Primary Care Pediatricians (PCPs). This highlights the urgent need for a culturally appropriate, psychometrically validated tool tailored to the Italian paediatric healthcare setting. In response, we developed the CLAP (Comunicazione e Linguaggio in Ambulatorio Pediatrico) screening tool: an innovative, age‐specific instrument designed to facilitate the early identification of children aged 24 to 72 months at risk of clinically relevant language difficulties, including those who may require further assessment for DLD. CLAP was intended to provide PCPs with a quick, non‐invasive, and easy‐to‐administer instrument for routine well‐child visits (Barry et al. 2024; Holzinger et al. 2021; Speights et al. 2024). The tool provides clear scoring procedures, whereas CLAP‐specific clinical implementation and follow‐up pathways will require further development and validation in subsequent phases of the project. The selection of items was informed by a review of the literature on early linguistic risk indicators, screening procedures used in paediatric primary care, and the characteristics of language development in both monolingual and bilingual populations (So and To 2022). Early indicators consistently reported in the literature include delays in gesture production, limited expressive and receptive vocabulary, morphosyntactic difficulties, absence of early word combinations—that is, the child's ability to combine two or more words to form simple sentences—and phonological or articulation deficits. Additional risk factors such as male sex, prematurity, twin birth, and family history of language disorders were also taken into account.

In parallel, the methodological framework guiding the development of the tool was grounded in both Classical Test Theory (CTT) and more recent psychometric approaches, particularly Item Response Theory (IRT). Advances in these methods have improved instrument development by enhancing item discrimination and measurement precision across a range of abilities. Because screening tools must be both psychometrically sound and clinically informative, relying on a single analytic framework may be insufficient. CTT is useful for evaluating overall test performance, Confirmatory Factor Analysis (CFA) for examining the instrument's internal structure, and IRT for assessing how well individual items discriminate across different levels of language ability, especially among children at greater risk for language disorders. These approaches are therefore complementary and particularly suitable for the development and preliminary validation of a screening tool for use in primary care (Tsui et al. 2024; Campo‐Arias and Oviedo 2008).

Accordingly, the current pilot study aimed to evaluate the psychometric properties of the CLAP instrument using a multi‐method approach combining CTT, CFA, and IRT, and to examine its accuracy across age groups.

## 2 Methods

### 2.1 Development of the CLAP Screening Tool

The development of the CLAP screening tool was conducted in multiple phases. Initially, a literature review was carried out to identify validated instruments for the early detection of language disorders (unpublished data, University of Torino, Speech Therapy Degree Program).

Subsequently, a multidisciplinary expert panel consisting of five speech‐language pathologists (SLPs) and four paediatricians contributed to the initial draft of the tool. CLAP was developed as a set of age‐specific screening versions, each aligned with research‐supported developmental milestones, focusing on the early detection of DLDs in children aged 24–72 months. Children under 24 months were excluded due to high variability in language development. The chosen age groups (24–30, 36–42, 48–54 months) were deliberately aligned with the standard well‐child visit schedule. Some intermediate months are therefore not directly covered, but this is not a practical issue, as those months typically do not correspond to scheduled paediatric visits for otherwise healthy children. This approach allows for language screening to be incorporated into routine care and may improve family adherence by avoiding an additional screening appointment. An additional version for children aged 60–72 months was tested to support the identification of late‐detected language difficulties among children who may have missed earlier well‐child visits, which in Italy are not scheduled in this age range (e.g., adopted children or children from recently immigrated families). This recognizes the ongoing importance of monitoring language development at this age.

Each age‐specific CLAP version includes a structured set of items along with contextual questions about the child's age and sex, twin birth, bilingualism, native language, school attendance, and behavioural observations during the visit. Demographic and basic medical history data are also collected. Items may involve parental reports, paediatrician observations, or brief language tasks and are designed to explore multiple domains of early communicative and linguistic development that are known to be important predictors of DLD. Specifically, the items evaluate: (1) early communicative behaviours, including gesture production and communicative intent; (2) lexical development, focusing on both receptive and expressive vocabulary; (3) morphosyntactic skills, especially the appearance of word combinations and basic sentence structures; (4) phonological and articulatory development. The relationship between the CLAP items and the linguistic domains studied is summarized in Table S1.

Responses are in a binary format (YES/NO), with “NO” indicating an incorrect response to the corresponding item. The total score is determined by counting the number of “NO” responses.

The current version of the CLAP includes four age‐specific tools for the 24–30‐, 36–42‐, 48–54‐, and 60–72‐month age ranges (Supplementary Materials S2, Italian Version, and S3, English Version).

### 2.2 Primary Care Paediatricians Enrolment

Overall, 33 PCPs affiliated with the Piedmont Regional Section of the Italian Society of Pediatric Primary Care (SiCuPP) received training in CLAP administration and data collection during a dedicated workshop at the Mauriziano Hospital in Torino, Italy. Of these, 23 actively participated in the study. Paediatricians were instructed to administer the CLAP tool during routine well‐child visits.

### 2.3 Children's Enrolment and Sample Size

Children were enrolled consecutively and prospectively from January to April 2025. Written informed consent was obtained from parents or legal guardians before participation. This pilot study aimed to enrol 200 children (50 participants per age group), and recruitment was planned to stop once the target sample size was reached (Kunselman 2024). Exclusion criteria included known sensory or neurodevelopmental disorders, current or previous speech‐language therapy, and insufficient exposure to Italian, defined as having arrived in Italy <12 months before enrolment and lacking regular exposure to Italian either at home or in community settings (e.g., nursery or preschool) during that period, to avoid misclassifying limited language exposure as a potential language disorder.

### 2.4 Procedure

Paediatricians entered scores into REDCap (Research Electronic Data Capture), a secure, web‐based platform for electronic data collection and management in clinical and research settings, accessible only to authorized users with a username and password (Harris et al. 2019). In REDCap, each participant was assigned an anonymized ID. Speech‐language evaluations were scheduled within 30 days.

### 2.5 Speech‐Language Evaluation

Evaluations lasting about 20 min were conducted by four experienced SLPs either at the Mauriziano Hospital or online. Sessions were recorded for later review. The assessment protocols included: Phonemic repetition plus PVB (Primo Vocabolario del Bambino, based on Italian MB‐CDI) for children aged 24–36 months (Caselli et al. 2016); and Phonemic repetition combined with a picture description task from TVL (Test di Valutazione del Linguaggio) for children aged 37–72 months (Cianchetti and Sannio Fancello 1997). Specifically, the Phonemic repetition tasks required repeating words and nonwords containing Italian phonemes in different word positions (initial, medial, and within consonant clusters); the PVB collected vocabulary data from caregivers; and the picture description tasks assessed spontaneous speech. Based on test scores, the child was classified as “pathological” if performance on at least one test was below −2 standard deviations or the 5th percentile; as “to be reviewed” if scores were between −1.5 and −2 standard deviations or between the 5th and 10th percentile on at least one test; and as “within normal limits” in all other cases.

SLPs were blinded to CLAP scores. Families received a participation certificate at the end of the speech‐language evaluation, and were informed that the evaluation outcome would be shared with their primary care paediatrician. They were then referred back to their paediatrician for further clinical management. Children in the pathological subgroup were immediately referred for specialist evaluation, and, when appropriate, access to speech‐language therapy services within the local healthcare system was recommended. Children in the to‐be‐reviewed subgroup were monitored in routine primary care by their paediatrician, who could track language development over time and decide whether referral for a formal speech–language evaluation was necessary. This approach aligns with the CLAP's intended role as a primary care screening tool.

### 2.6 Statistical Analysis and Validation Process

#### 2.6.1 CTT

To evaluate internal consistency reliability, the Kuder‐Richardson Formula 20 (KR‐20) was used. This dichotomous‐item coefficient was calculated separately for each age group to assess the test's homogeneity. A value of 0.70 or higher was deemed acceptable, in line with standard thresholds for internal consistency reliability (Campo‐Arias and Oviedo 2008).

#### 2.6.2 Structural Validity

Structural validity was examined using CFA, which tested the hypothesized factorial structure of the screening tool and verified the model's adequacy before proceeding with IRT analyses. Before conducting CFA, the data's suitability for factor analysis was evaluated by calculating the Kaiser‐Meyer‐Olkin (KMO) measure of sampling adequacy, both overall and at the item level. The KMO statistic, which ranges from 0 to 1, measures the proportion of variance among variables attributable to common factors. It indicates the strength of the partial correlations and how well the other variables explain each variable in the model. A KMO value above 0.80 is generally considered excellent sampling adequacy, with values ≥ 0.60 typically regarded as acceptable. Model fit was then assessed using multiple indices recommended in Structural Equation Modeling. The Comparative Fit Index (CFI) was used to evaluate how well the model fit compared to a baseline (null) model; values of 0.90 or higher were considered indicative of a good fit. The Tucker‐Lewis Index (TLI), which penalizes excessive model complexity, was also examined; values above 0.95 indicate an excellent fit. For the Root Mean Square Error of Approximation (RMSEA), which considers model parsimony, values below 0.08 were considered acceptable, and those under 0.06 indicated a close fit. Finally, the Standardized Root Mean Square Residual (SRMR), reflecting the average standardized difference between observed and predicted correlations, was evaluated. Values below 0.08 indicated an adequate fit. Together, these indicators provided a thorough evaluation of how well the hypothesized factor structure aligned with the observed data (Charrier et al. 2025).

#### 2.6.3 IRT

Item‐level psychometric properties were further examined using IRT. For each cohort, two logistic models were tested: the one‐parameter logistic model (1PL or Rasch) and the two‐parameter logistic model (2PL). The most appropriate model was chosen based on fit indices, including the log‐likelihood, Akaike Information Criterion (AIC), Bayesian Information Criterion (BIC), and sample‐size‐adjusted BIC (SABIC). All models assumed a Gaussian distribution for the latent trait, and parameters were estimated using the Expectation‐Maximization (EM) algorithm.

#### 2.6.4 Item Information Functions (IIF)

To assess how much information each item contributed across the range of the latent trait (*θ*), IIFs were calculated for all models and groups. IIFs show an item's measurement accuracy at different ability levels and are based on item parameters estimated by IRT models. For each item, the IIF was plotted to identify the *θ* levels where the item was most informative. These curves provided a visual way to compare items, allowing us to distinguish those that offered precise measurements within specific trait ranges from those that offered little information across the continuum. This analysis was crucial for evaluating item quality, spotting poorly performing items, and guiding item selection to enhance the scale.

#### 2.6.5 ROC Curve Analysis

To evaluate the model's performance across different age groups, Receiver Operating Characteristic (ROC) curves were generated for each group. The area under each ROC curve (AUC) was calculated as a summary measure of overall discriminative ability. For each ROC curve, sensitivity (SE) and specificity (SP) were measured at multiple thresholds, and the median SE and SP were extracted for comparison. Thresholds of 0.5, 1.5, and 2.5 were tested in each group, with the 2.5th, 50th, and 97.5th percentiles considered for comparison. Candidate thresholds were identified through ROC analysis, selecting the best balance between SE and SP, with a preference for higher sensitivity to detect children at risk of DLD early, enabling further assessment and reducing missed positive cases. ROC analyses were performed both before and after an item‐level analysis using Factor Analysis (FA). This process aimed to refine the item set by removing questions that did not significantly contribute to the underlying construct. Items were excluded based on a combination of statistical criteria from FA and clinical judgment, ensuring that the removal aligned with expert evaluation and preserved clinical relevance.

Analyses were performed using R software version 4.5.2 with the psych (version 2.6.1), lavaan (version 0.6‐21), validate (version 1.1.7), and mirt (version 1.45.1) packages (R Core Team 2022).

## 3 Results

### 3.1 Enrolled Participants

The pilot phase of the CLAP study aimed to include a final sample of 200 children, divided into four age‐specific groups of 50 participants each (24–30, 36–42, 48–54, and 60–72 months). Only children who completed the full evaluation pathway—CLAP administration by PCPs followed by an independent assessment by an SLP—were included in the analyses. After reaching the initial target of 200 enrolled children, 45 did not complete the SLP assessment and were therefore excluded. Recruitment was then resumed to replace these cases, resulting in a final analytic sample of 200 children with complete data.

Overall, 24% of the sample fell into the pathological scoring group, defined as children whose language status was rated “pathological” by a speech‐language pathologist. This proportion was 24.0% (12/50) in the 24–30‐month group, 34.0% (17/50) in the 36–42‐month group, 24.0% (12/50) in the 48–54‐month group, and 10.0% (5/50) in the 60–72‐month group.

### 3.2 CTT

#### 3.2.1 Reliability

The results demonstrated acceptable internal consistency for the 24–30‐ and 36–42‐month groups (KR‐20 = 0.695 and 0.777, respectively), while lower reliability was found in the older age groups (KR‐20 = 0.504 and 0.113 for the 48–54‐ and 60–72‐month groups, respectively).

#### 3.2.2 Structural Validity

Table 1 presents the results of CFA performed separately for each age group to assess structural validity. The aim was to evaluate how well the hypothesized unidimensional structure of the screening tool fits by analyzing item loadings on a single latent factor (LF1), along with standard fit indices and sampling adequacy measures. Overall, sampling adequacy was satisfactory across most age groups (KMO ≥ 0.60), except for the 60–72‐month group, which had a very low KMO (0.50).

**TABLE 1:** Confirmatory factor analysis (CFA) results by age group, including standardized item loadings and overall model fit indices.

Age groups	Items	Loading	CFI	TLI	RMSEA	SRMR
24–30 months	Item 1	0.591	0.921	0.841	0.122	0.057
	Item 2	0.629				
	Item 3	0.794				
	Item 4	0.044				
	Item 5	0.661				
36–42 months	Item 1	0.822	0.896	0.689	0.277	0.064
	Item 2	0.915				
	Item 3	0.603				
	Item 4	0.443				
48–54 months	Item 1	0.626	1	1.481	0	0.015
	Item 3	0.694				
	Item 4	0.131				
	Item 5	0.426				
60–72 months	Item 2	0.338	1	1	0	0
	Item 3	−0.011				
	Item 4	0.490				

*Note: Loading* refers to the standardized factor loading of each item on the single latent factor (LF1), indicating the strength and direction of the association between the item and the latent construct.

Reference cut‐offs for fit indices:

CFI (Comparative Fit Index): ≥ 0.9 (acceptable); ≥ 0.95 (good)

TLI (Tucker‐Lewis index): ≥ 0.9 (acceptable); ≥ 0.95 (good)

RMSEA (Root Mean Square Error of Approximation): *≤* 0.08 (acceptable); *≤* 0.05 (good)

SRMR (Standardized Root Mean Square Residual): *≤* 0.08 (acceptable); *≤* 0.05 (good)

For the 24–30‐month group, five items were included. The CFA generally supported the unidimensional model (LF1), with mostly moderate‐to‐strong item loadings (0.794, 0.661, 0.629, and 0.591 for Items 3, 5, 2, and 1, respectively) and acceptable fit indices (Table 1). One item (Item_4) showed a negligible loading and poor item‐level sampling adequacy (item‐specific KMO = 0.441), consistent with a limited contribution to the latent construct.

In the 36–42‐month group, four items were analyzed. Item loadings were generally strong (Item_1 and Item_2) to moderate (Item_3 and Item_4) (Table 1). Model fit was mixed, with acceptable CFI (0.896) and SRMR (0.064), but poorer TLI (0.689) and elevated RMSEA (0.277).

In the 48–54‐month group, five items were analyzed. One item (Item_2) was excluded due to a lack of variance (all responses were “yes”) and therefore lacked discriminative value. The remaining four items mostly showed adequate loadings, while Item_4 again performed weakly (0.131). Fit indices indicated a good model fit (Table 1).

In the 60–72‐month group, four items were analyzed. One item (Item_1) was excluded due to a lack of variance (all “yes” responses). The remaining three item loadings were weak, including a slightly negative loading for Item_3 (−0.011). Because this value was very close to zero, it should not be interpreted as a meaningful inverse relationship with the latent construct, but rather as indicating that Item_3 did not contribute to the common factor. Together with the low KMO value, this indicates limited data adequacy for this age group, despite apparently excellent fit indices.

#### 3.2.3 IRT

IRT models were applied to the four age groups to evaluate item characteristics and model fit using one‐parameter (1PL) and two‐parameter (2PL) logistic models (Table 2). Model fit was assessed using log‐likelihood, AIC, BIC, and SABIC criteria.

**TABLE 2:** Item Response Theory (IRT) parameters across age groups, showing the selected logistic model and item discrimination and difficulty estimates.

Age groups	Logistic model from IRT	Items	Discrimination (a)	Difficulty (b)
24–30 months	two‐parameter logistic model (2PL)	Item 1	2.35	−1.24
		Item 2	22.79	−1.39
		Item 3	5.29	−0.76
		Item 4	0.33	−7.59
		Item 5	27.24	−0.01
36–42 months		Item 1	13.22	−1.07
		Item 2	25.19	−0.78
		Item 3	2.27	−0.81
		Item 4	1.45	−0.99
48–54 months	one‐parameter logistic model (1PL)	Item 1	—	−1.82
		Item 3	—	−2.86
		Item 4	—	−1.98
		Item 5	—	0.00
60–72 months		Item 2	—	−3.15
		Item 3	—	−0.80
		Item 4	—	−2.33

In the 24–30‐month group, the 2PL model demonstrated a better fit than the 1PL model (AIC = 196.46 vs. 212.65; SABIC = 184.19). The parameters of the 2PL model showed high discrimination for items 2 and 5 (*a* = 22.79 and 27.24, respectively), and very low difficulty for Item_4 (*b* = −7.59).

In the 36–42‐month group, the best‐fitting model was the 2PL model, with lower AIC and SABIC values (AIC = 175.16; SABIC = 165.34) than the 1PL model. Discrimination was highest for Item_2 (*a* = 25.19), and all items showed similar difficulty levels, around −0.8 to −1.0.

In the 48–54‐month group, the model fit indices slightly favoured the 1PL model (AIC = 188.26; SABIC = 183.35), although the differences were minimal. Convergence warnings occurred for the 2PL model due to early termination of the EM cycle. The 1PL model estimates indicated moderate‐to‐high difficulty levels across items.

Finally, in the 60–72‐month group, the 1PL model demonstrated a better fit (AIC = 129.86; SABIC = 126.18). Item difficulty values ranged from −3.15 to −0.80.

#### 3.2.4 Item Information Functions (IIF)

**FIGURE 1:**
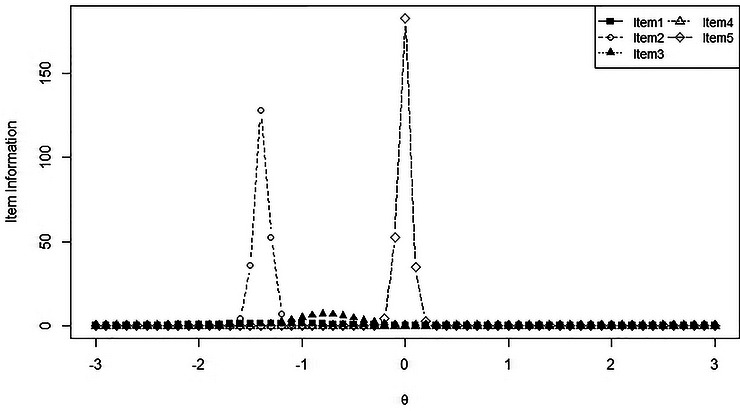
Item Information Functions (IIFs) from the 2PL model for Items 1–5 across the latent trait continuum (*θ*) in the 24–30‐month age group.

**FIGURE 2:**
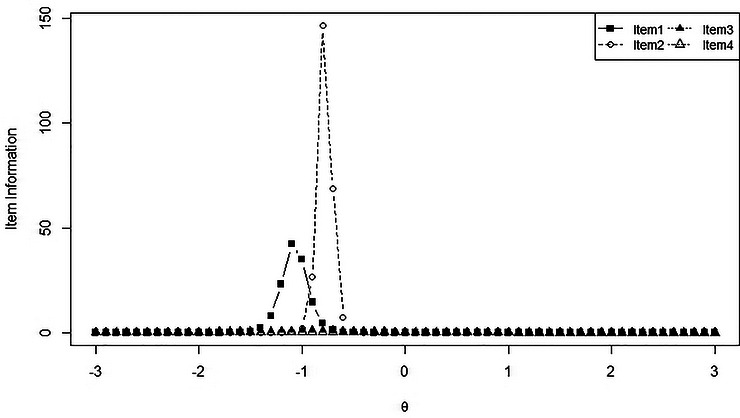
Item Information Functions (IIFs) from the 2PL model for Items 1–4 across the latent trait continuum (*θ*) in the 36–42‐month age group.

**FIGURE 3:**
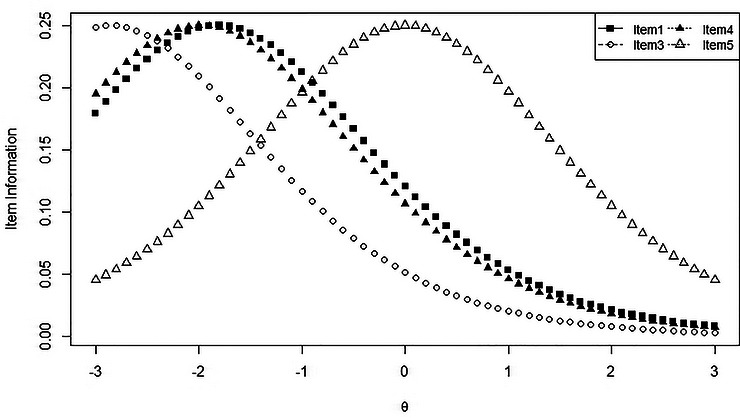
Item Information Functions (IIFs) from the 1PL model for Items 1, 3, 4, and 5 across the latent trait continuum (*θ*) in the 48–54‐month age group.

**FIGURE 4:**
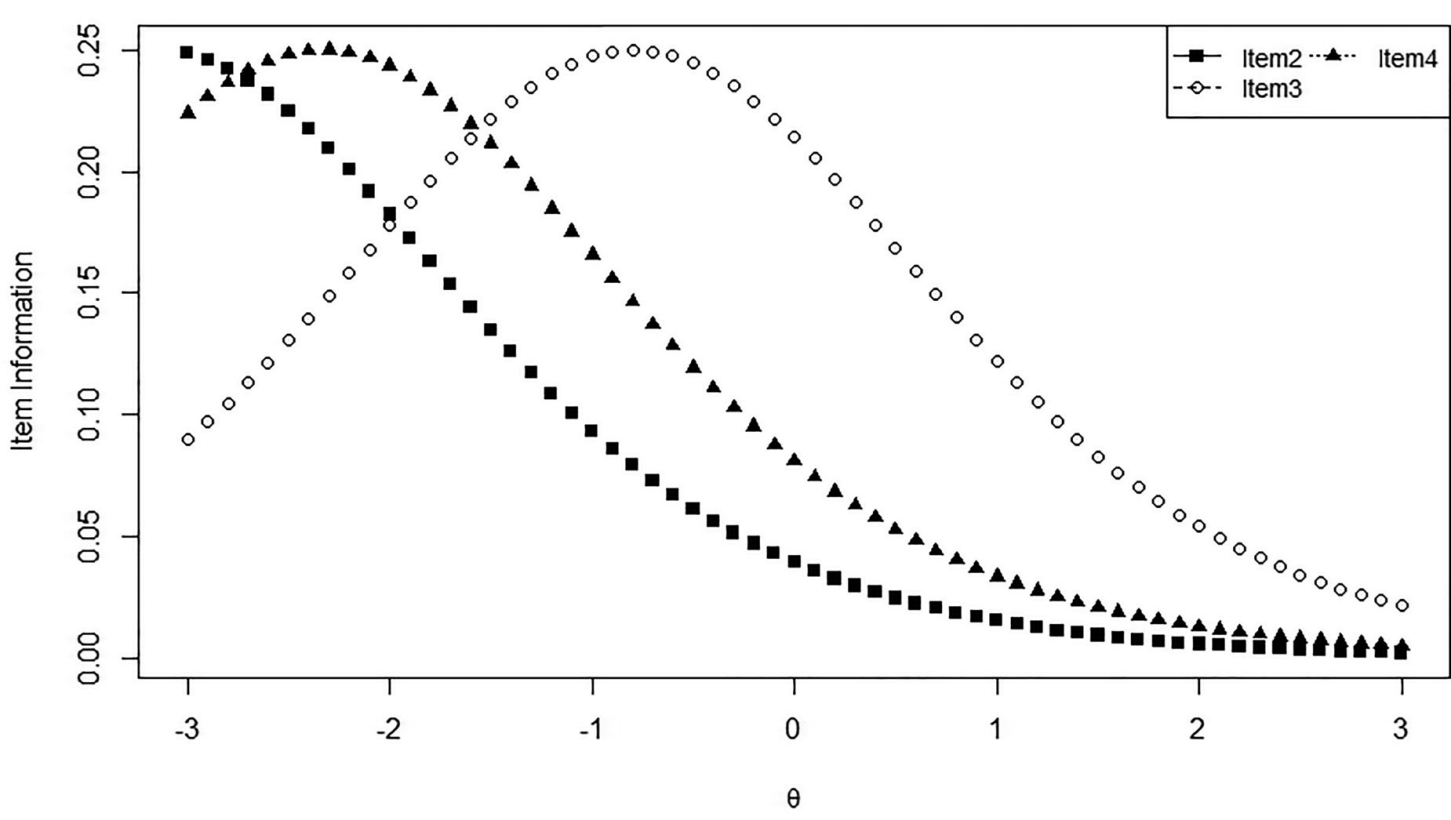
Item Information Functions (IIFs) from the 1PL model for Items 2–4 across the latent trait continuum (*θ*) in the 60–72‐month age group.

To assess the accuracy and contribution of each item across different levels of the latent trait (*θ*), we examined the IIFs for various subsets of items (Figures 1, 2, 3, 4). In each figure, the *x*‐axis shows the latent trait level (*θ*), and the *y*‐axis indicates the amount of item information, with higher values reflecting greater measurement precision at a specific trait level (Figures 1, 2, 3, 4 use a different scale on the *y*‐axis because the information comes from the 2PL model for Figures 1, 2 and from the 1PL model for Figures 3, 4).

In the first model (Figure 1, age group 24–30 months), Item_5 provided the most information, peaking sharply around *θ* = 0, indicating that it is highly informative for respondents with average levels of the latent trait. Item_2 also showed a narrower peak centred at *θ* = −1.5, highlighting its discriminative ability among respondents with lower trait levels. Items 1 and 3 offered moderate information, while Item_4 contributed virtually no information across the trait continuum, confirming previous findings from factor analysis.

The second model (Figure 2, age group 36–42 months) showed a similar pattern, with Item_2 providing the most information and a sharp focus around *θ* = −1. Item_1 had a smaller peak in the same area, while Items 3 and 4 contributed little, indicating lower discriminative power.

The third model (Figure 3, age group 48–54 months), which displayed a more balanced curve distribution, showed that all items offered relatively broad but low information, with Item_5 peaking near *θ* = 0 and Items 1 and 4 peaking around *θ* = −1.5 to −2. This pattern indicates that, collectively, the items can assess a wide range of *θ* levels, albeit with limited precision.

In the fourth model (Figure 4, age group 60–72 months), information was more evenly distributed across the lower end of the *θ* spectrum. Item_4 was most informative around *θ* = −2, and Item_2 provided information for very low levels of *θ* (around −2.5). Overall, none of the items offered substantial information at medium or high levels of the latent trait.

#### 3.2.5 ROC Curve Analysis Before and After Factor Analysis

We assessed the predictive model's performance across the four age groups by examining sensitivity and specificity at selected thresholds, both before and after factor‐analysis‐based item reduction (Table 3).

**TABLE 3:** Sensitivity and specificity (50th percentile) at three score thresholds before and after factor‐analysis‐based item reduction, by age group.

24–30‐month group				
Threshold	SE before	SP before	SE after	SP after
0.50	1.00	0.63	1.00	0.63
**1.50**	0.92	0.87	**0.92**	**0.87**
2.50	0.50	0.95	0.50	0.95

36–42‐month group				
Threshold	SE before	SP before	SE after	SP after
**0.50**	0.67	0.72	**0.67**	**0.72**
1.50	0.50	0.91	0.50	0.91
2.50	0.33	1.00	0.33	1.00

48–54‐month group				
Threshold	SE before	SP before	SE after	SP after
**0.50**	0.83	0.50	**0.83**	**0.55**
1.50	0.50	0.84	0.33	0.89
2.50	0.25	0.95	0.08	0.95

60–72‐month group				
Threshold^a^	SE before	SP before	SE after	SP after
0.50	0.40	0.56	0.00	0.82
1.50	0.00	0.91	0.00	0.98
2.50	0.00	1.00	0.00	1.00

*Note*: Selected thresholds and corresponding sensitivity and specificity values are shown in **bold**.

^a^No clinically acceptable cut‐off.

As part of this process, specific items were removed based on both statistical and clinical considerations. Item_2 in the 48–54 months group and Item_1 in the 60–72 months group were excluded because they received uniform responses (“yes” in all cases), indicating they lack discriminative value. These adjustments were intended to improve the scale's construct validity while preserving clinical interpretability.

Age‐stratified ROC analyses were then used to identify the optimal cut‐offs for the final item set. Cut‐offs were selected to achieve the best balance between sensitivity and specificity, with priority given to sensitivity because the CLAP is intended as a screening tool. For the 24–30‐month group, with an AUC of 0.93, the selected cut‐off was 1.5, corresponding to a sensitivity of 0.92 and a specificity of 0.87. For the 36–42‐month group (AUC of 0.75), the selected cut‐off was 0.5, with a sensitivity of 0.67 and a specificity of 0.72. For the 48–54‐month group (AUC of 0.74), the selected cut‐off was 0.5, with a sensitivity of 0.83 and a specificity of 0.55. For the 60–72‐month group, diagnostic accuracy was poor (AUC of 0.46), and no clinically acceptable cut‐off could be identified because sensitivity dropped to 0 after item reduction. Therefore, the current 60–72‐month version should not be used as a standalone screening tool without substantial revision.

## 4 Discussion

This pilot study aimed to assess the psychometric properties and accuracy of the CLAP screening tool for early detection of children at risk of DLD in Italian primary care, using a rigorous multi‐method statistical approach.

Overall, CLAP demonstrated acceptable psychometric properties across most age groups, with better performance in younger children. Internal consistency, assessed using KR‐20, was satisfactory in the younger groups, while lower reliability was observed in the older groups. CFA supported the tool's structural validity in younger children, identifying a largely unidimensional construct with a good model fit, although some items showed weaker loadings. IRT analyses further confirmed the effectiveness of the 24‐ to 42‐month versions, showing good discrimination and informativeness for several items, whereas the versions designed for older children were more affected by ceiling effects and less informative. The IIF indicated that the most informative items provided greater precision in the lower‐to‐average range of language ability, aligning with CLAP's purpose as an early screening tool. Together, these findings support the instrument's psychometric value in early childhood and offer useful guidance for future item refinement.

These findings are consistent with previous work showing that brief language screening instruments can be feasible and clinically useful in primary care settings, particularly when they are age‐specific and compatible with routine child health visits (Law and Harris 2000; Lavesson et al. 2018; Holzinger et al. 2021; Visser‐Bochane et al. 2021). At the same time, our results confirm that screening accuracy may vary substantially by age and item content, highlighting the importance of age‐specific validation rather than assuming uniform performance across the preschool period.

In the youngest age group, CLAP performed particularly well, aligning with its clinical goal of supporting early identification of children who may have language difficulties, including both children at risk of DLD and late talkers. Early identification is particularly relevant in this age range because early expressive delays may either be a temporary developmental lag or the first sign of more persistent language difficulties. This developmental variability complicates clinical decision‐making and underscores the need for structured screening tools to guide monitoring and referral decisions in primary care (Sax and Weston 2007; Paul et al. 2018; Gard et al. 1993; Snowling et al. 2016; Sim et al. 2019). In the 36‐ to 42‐month and 48‐ to 54‐month groups, IRT analyses also indicated generally adequate item calibration and discrimination.

In contrast, the 60‐ to 72‐month version demonstrated limited diagnostic accuracy. Most items were too simple for children in this age group, and only three could be meaningfully included in the IRT analysis. Several items failed to accurately distinguish between children with and without clinically significant language concerns, leading to a relatively high false‐positive rate. This finding is consistent with the developmental rationale underlying age‐specific screening: screening items must be sufficiently aligned with the expected linguistic abilities and developmental demands of the target age group. In older pre‐schoolers, items that are not sufficiently challenging may exhibit ceiling effects and lose their discriminative value, even when included in an age‐specific version. Similar concerns have been raised in previous studies and reviews, which emphasize that screening tools must be closely aligned with the developmental level and linguistic demands of the target population (Wallace et al. 2015; So and To 2022). While the tool may still have some clinical value in identifying children with previously unrecognized developmental issues, its current psychometric performance does not support reliable standalone use in this age range. These findings indicate that the 60‐ to 72‐month version requires substantial revision before it can be broadly implemented, and suggest that a different screening method or a specifically designed instrument might be more suitable for children in this age group.

Finally, the diagnostic accuracy results should be interpreted in relation to CLAP's intended use as a first‐level screening tool in primary care. The 24–30‐month version showed the strongest and most clinically interpretable performance, with excellent discrimination (AUC = 0.93) and a selected cut‐off of 1.5, corresponding to good sensitivity and specificity. The 36–42‐ and 48–54‐month versions showed fair discrimination. However, the selected cut‐off was 0.5 in both groups. This threshold identifies children with very low performance on the retained items, suggesting that these versions may be useful for detecting more evident language difficulties but may be less sensitive to milder or borderline profiles. The 60–72‐month version showed poor diagnostic accuracy, and no clinically acceptable cut‐off could be identified, indicating the need for substantial revision before standalone use.

In our sample, 24% of children were classified as having clinically relevant language difficulties based on the follow‐up SLP assessment. This figure should not be interpreted as the prevalence of DLD, but rather as the proportion of children identified as requiring further clinical attention within this pilot screening context. This group likely includes not only children who may meet criteria for DLD, but also children with broader patterns of atypical language development that require further diagnostic evaluation. The relatively high percentage observed may reflect possible self‐selection bias, as families more concerned about their child's language development may have been more likely to participate, as well as the broader clinical outcome adopted in this pilot phase. From a screening standpoint, this aligns with CLAP's intended purpose: to identify children who need more detailed assessment rather than to provide a definitive diagnosis.

## 5 Strengths and Limitations

This study has several strengths. It provides the first pilot validation of an age‐specific, brief, and clinically feasible screening tool designed for use in Italian primary care paediatric settings. All children underwent an independent speech–language assessment, and analyses were conducted separately by age group using a multi‐method psychometric approach, including internal consistency, CFA, IRT, item information functions, and ROC analyses. This allowed us to evaluate both overall test performance and item‐level functioning, providing useful guidance for future refinement of the tool.

Some limitations should also be acknowledged. First, this was a pilot study with relatively small sample sizes within each age group, and the findings therefore require confirmation in a larger, more representative population‐based sample. For the same reason, background variables such as multilingual exposure and anamnestic factors were collected but not analyzed in relation to CLAP performance, as the pilot sample and the number of events were not sufficient for meaningful subgroup analyses. Their influence on screening outcomes should therefore be examined in larger validation studies. Second, the relatively high proportion of children classified as having clinically relevant language difficulties may limit the generalizability of the estimates and should be considered when interpreting the diagnostic accuracy estimates. Third, although the selection of cut‐offs was guided by CLAP's intended role as a first‐level screening tool, the clinical interpretation of these thresholds requires caution. In particular, the selected cut‐off of 0.5 in the 36–42‐ and 48–54‐month groups identifies children with very low performance on the retained items, suggesting that these versions may be more effective in detecting more evident language difficulties than milder or borderline profiles. Conversely, prioritizing sensitivity in a screening context may reduce specificity and increase false‐positive results, with potential implications for unnecessary referrals and burden on families and speech–language services. Finally, the 60–72‐month version showed limited psychometric and diagnostic performance, indicating that it requires substantial revision before routine use.

## 6 Conclusion

CLAP is a brief, age‐specific screening tool that has shown promising psychometric properties for identifying children at risk of clinically relevant language difficulties, including those who may require further assessment for DLD. Diagnostic accuracy was good to fair up to 54 months, with the strongest performance observed in the 24–30‐month group. This supports timely referral for further diagnostic assessment and appropriate speech–language evaluation. Its brief format, rapid administration, and intended compatibility with routine well‐child visits support its potential for adoption in primary care. However, performance declines in children older than 60 months, underscoring the need for targeted item revision to better capture language difficulties in older pre‐schoolers. Although findings are promising, broader validation in more representative populations is underway to confirm external validity. A larger validation and implementation phase is being planned, and a parallel survey of primary care paediatricians is currently assessing usability, time efficiency, and perceived clinical utility to guide future implementation within national paediatric care pathways.

## Funding

The authors have nothing to report

## Ethics Statement

The study was approved by the Local Ethical Research Committee in January 2025 (Protocol Number 0000095).

## Conflicts of Interest

The authors declare no conflicts of interest.

## Supporting information




**Supplementary Table S1**: Linguistic domains assessed by the CLAP screening items.

**Supplementary Information**: jlcd70282‐supp‐0002‐SuppMat.pdf

**Supplementary Information**: jlcd70282‐supp‐0003‐SuppMat.pdf

## Data Availability

The data (de‐identified participant data) supporting the findings of this study are available from the corresponding author, LC, upon reasonable request. However, according to Italian law, data may not be analyzed for purposes other than those approved by the Institutional Review Board.
