# Supplementary material for: Early Identification of DLD in Paediatric Practice: A Pilot Validation of the CLAP Screening Tool in Italian Outpatient Settings
Source: Int J Lang Commun Disord. 2026 Jun 25;61(4):e70282. doi: 10.1111/1460-6984.70282 (PMC13297869; doi:10.1111/1460-6984.70282)
Supplement: Supplementary file 1 — Supplementary Table S1: Linguistic domains assessed by the CLAP screening items. [file JLCD-61-0-s003.docx]

**S1. Supplementary Table – Linguistic domains assessed by the CLAP screening items**

| **Age range** | **CLAP Item**  **(short description)** | **Type of task** | **Linguistic domain assessed** | **Item #** |
| --- | --- | --- | --- | --- |
| **24-30 months** | Child easily understood by family | Parent report | Speech intelligibility / phonological development | **1** |
|  | Produces at least 15 everyday words | Parent report | Expressive vocabulary | **2** |
|  | Produces two-word combinations | Parent report | Early morphosyntactic development | **3** |
|  | Understands simple command (“Take…”) | Direct assessment | Receptive language comprehension | **4** |
|  | Names objects in pictures (correct production of target sounds) | Direct assessment | Expressive vocabulary/ phonological-articulatory development | **5** |
| **36-42 months** | Child understood by unfamiliar listeners | Parent report | Speech intelligibility | **1** |
|  | Pediatrician understands child | Clinical observation | Functional communicative intelligibility | **2** |
|  | Naming objects in pictures (production of target phonemes) | Direct assessment | Expressive vocabulary/ phonological-articulatory development | **3** |
|  | Describing actions in pictures (verb use, multi-word utterances) | Direct assessment | Expressive morphosyntax | **4** |
| **48-54 months** | Child understood by unfamiliar listeners | Parent report | Speech intelligibility | **1** |
|  | Production of complete sentences | Parent report | Morphosyntactic development | 2 |
|  | Pediatrician understands child | Clinical observation | Functional communicative intelligibility | **3** |
|  | Sentence comprehension (spatial relation task) | Direct assessment | Receptive morphosyntax / grammatical comprehension | **4** |
|  | Naming objects in pictures (production of consonant clusters) | Direct assessment | Expressive vocabulary/ phonological-articulatory development | **5** |
| **60-72 months** | Pediatrician understands child | Clinical observation | Functional speech intelligibility | 1 |
|  | Sentence comprehension task | Direct assessment | Receptive language | **2** |
|  | Naming objects and concepts (production of complex phonemes) | Direct assessment | Expressive vocabulary/phonological-articulatory development | **3** |
|  | Sentence repetition | Direct assessment | Morphosyntax and verbal working memory | **4** |

**Note.** **Bold** item# numbers indicate items analyzed after CFA.
