# Supplementary material for: Early Identification of DLD in Paediatric Practice: A Pilot Validation of the CLAP Screening Tool in Italian Outpatient Settings
Source: Int J Lang Commun Disord. 2026 Jun 25;61(4):e70282. doi: 10.1111/1460-6984.70282 (PMC13297869; doi:10.1111/1460-6984.70282)
Supplement: Supplementary file 2 — Supplementary Information: jlcd70282‐supp‐0002‐SuppMat.pdf [file JLCD-61-0-s001.pdf]

CLAP - Pediatra

Comunicazione e Linguaggio in Ambulatorio Pediatrico

UNIVERSITÀ DI TORINO

SICuPP

Nome e cognome:

Data di nascita:

Sesso: □ M □ F

Codice identificativo:

Data di compilazione:

24 – 30 mesi

□ Parto gemellare

□ Situazione di bilinguismo/multilinguismo
Lingua/e madre:

□ Frequenta l'asilo nido

Domande per i GENITORI	**Quando il bambino parla, viene compreso facilmente in famiglia?**	SI	NO
	**Dice almeno 15 parole di uso quotidiano (in qualsiasi lingua usata abitualmente)?*** Per esempio: mamma, papà, nonna, palla, bau bau, muu, casa, ecc. *Non importa come le dice, può anche avere una diversa pronuncia (es. "pappe" per "scarpe") o usare onomatopee (es. muu, brum brum).	SI	NO
	**Ha iniziato a dire brevi frasi?** Per esempio: "pappa più", "dà brum brum", "no c'è". Segnare NO se parla usando solo singole parole, es. "pappa", "dà", "più".	SI	NO
Domande per il BAMBINO	**Prendi… (la penna o la matita o la macchinina….)*** *Scegliere qualsiasi oggetto o gioco presente, purché facilmente riconoscibile dal bambino. Evitare di usare gesti che aiutino la comprensione. Segnare NO se il bambino non esegue il compito o ne esegue uno non richiesto/ errato.	SI	NO
	**Cosa c'è in queste immagini?** (**m**amma) *o* (**p**apà) *o* (**b**imbi) (**c**ane) (**g**atto) (**t**opo) [è sufficiente 1 parola fra le 3] 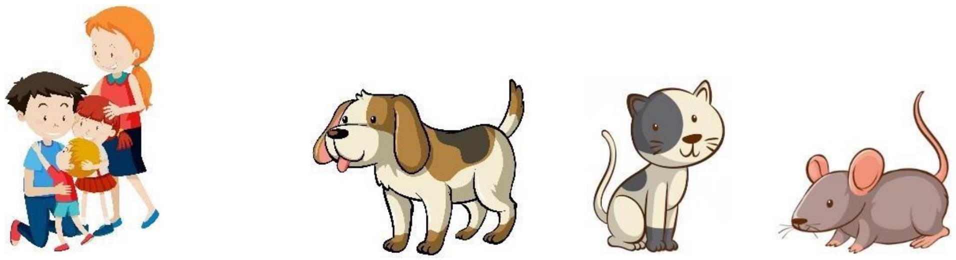 **Il bambino pronuncia bene almeno 3 delle letterine in grassetto?** Segnare NO se il bambino non dice nulla o se produce suoni non ben riconoscibili.	SI	NO

□ **IMPOSSIBILE COINVOLGERE IL BAMBINO**

TOTALE "NO": …./5

Progetto realizzato da Actis Maria e Bonzo Silvia (Logopediste - Università degli Studi di Torino)
In collaborazione con Dott.ssa E. Grosso (Logopedista), Dott.ssa G. De Cillis (Logopedista), Dott. A. Carpino (Pediatra)

CLAP - Pediatra

Comunicazione e Linguaggio in Ambulatorio Pediatrico

Nome e cognome:

Data di nascita:

Sesso: □ M □ F

Codice identificativo:

Data di compilazione:

36 – 42 mesi

□ Parto gemellare

□ Situazione di bilinguismo/multilinguismo
Lingua/e madre:

□ Frequenta la Scuola dell'Infanzia

GENITORI	**Quando il bambino parla, viene compreso facilmente dagli estranei?***<br>* Si intende persone che NON passano abitualmente la giornata con lui.	SI	NO
PEDIATRA	**Capisco il bambino quando mi parla?**<br>Segnare NO se il genitore deve ripetere o tradurre anche solo una parola	SI	NO
Domande per il BAMBINO	**Cosa c'è in queste immagini?**<br>(vaso) *o* (fiore) (ciuccio) (gelato) (sole)<br>[è sufficiente 1 parola fra le 2]<br>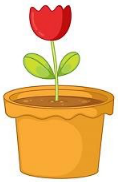 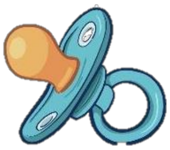 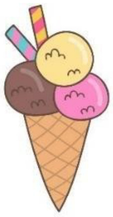 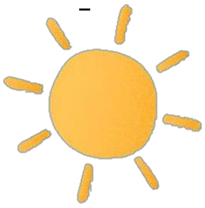<br>**Il bambino pronuncia bene almeno 3 delle letterine in grassetto?**<br>Segnare NO se il bambino dice ad esempio: "faso", "baso", "piole", "tutto", "zuzzo", "delato", "zelato", "tole", "ciole" …	SI	NO
	**Cosa fanno questi bimbi?**<br>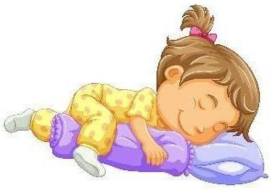 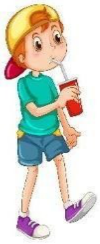 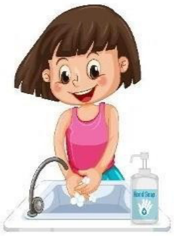<br>la bimba **dorme** il bimbo **beve** la coca-cola la bimba si **lava** le mani<br>**Il bambino descrive le immagini usando verbi e/o usando più parole?** Non importa *come* le dice.	SI	NO

□ **IMPOSSIBILE COINVOLGERE IL BAMBINO**

TOTALE "NO": …./4

Progetto realizzato da Actis Maria e Bonzo Silvia (Logopediste - Università degli Studi di Torino)
In collaborazione con Dott.ssa E. Grosso (Logopedista), Dott.ssa G. De Cillis (Logopedista), Dott. A. Carpino (Pediatra)

CLAP - Pediatra

Comunicazione e Linguaggio in Ambulatorio Pediatrico

Nome e cognome: Data di nascita: Sesso: ☐ M ☐ F

Codice identificativo:

Data di compilazione:

48 – 54 mesi

☐ Parto gemellare

☐ Situazione di bilinguismo/multilinguismo
Lingua/e madre:

☐ Frequenta la Scuola dell'Infanzia

GENITORI	**Quando il bambino parla, viene compreso facilmente dagli estranei?***<br>* Si intende persone che non passano abitualmente la giornata con lui.	SI	NO
GENITORI	**Ha iniziato a dire frasi complete (con articoli, preposizioni, ecc.)?**<br>Esempio: "voglio il gelato che mi piace tanto"; "il papà prende la bici del nonno".	SI	NO
PEDIATRA	**Capisco il bambino quando mi parla?**<br>Segnare NO se il genitore deve ripetere o tradurre anche solo una parola	SI	NO
Domande per il BAMBINO	**Qual è l'immagine corretta?**<br>**"il gatto è sulla scatola"**<br>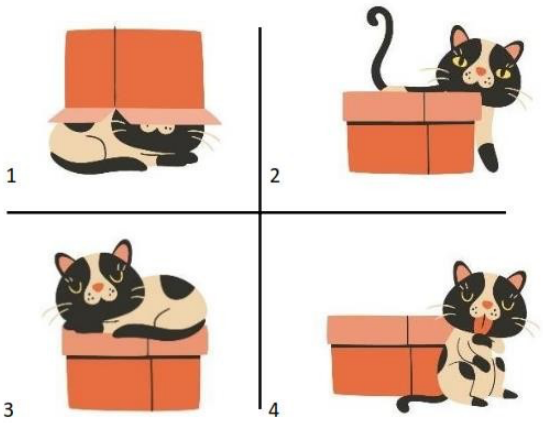 	SI	NO
Domande per il BAMBINO	**Cosa c'è in queste immagini?**<br>(**st**ella) (pa**st**a) (a**lb**ero) (fu**ng**o)<br>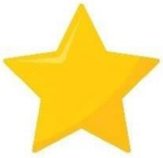 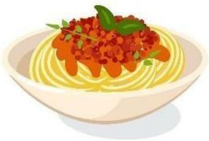 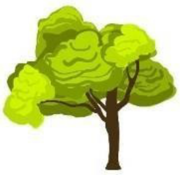 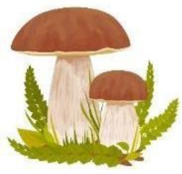<br>**Il bambino pronuncia bene almeno 3 dei gruppi di letterine in grassetto?**<br>Segnare NO se il bambino dice ad esempio: "tella", "sella", "patta", "passa", "abbero", "fuggo", …	SI	NO

☐ **IMPOSSIBILE COINVOLGERE IL BAMBINO**

TOTALE "NO": …./5

Progetto realizzato da Actis Maria e Bonzo Silvia (Logopediste - Università degli Studi di Torino)
In collaborazione con Dott.ssa E. Grosso (Logopedista), Dott.ssa G. De Cillis (Logopedista), Dott. A. Carpino (Pediatra)

CLAP - Pediatra

Comunicazione e Linguaggio in Ambulatorio Pediatrico

Nome e cognome: Data di nascita: Sesso: □ M □ F

Codice identificativo: Data di compilazione:

60 – 72 mesi

□ Parto gemellare

□ Situazione di bilinguismo/multilinguismo
Lingua/e madre:

□ Frequenta la Scuola dell'Infanzia
□ Frequenta la Scuola Primaria

PEDIATRA	**Capisco il bambino quando mi parla?**<br><br>Segnare NO se il genitore deve ripetere o tradurre anche solo una parola	SI	NO
Domande per il BAMBINO	**Qual è l'immagine corretta?**<br>**"ci sono una macchina lunga e una casa piccola"**<br>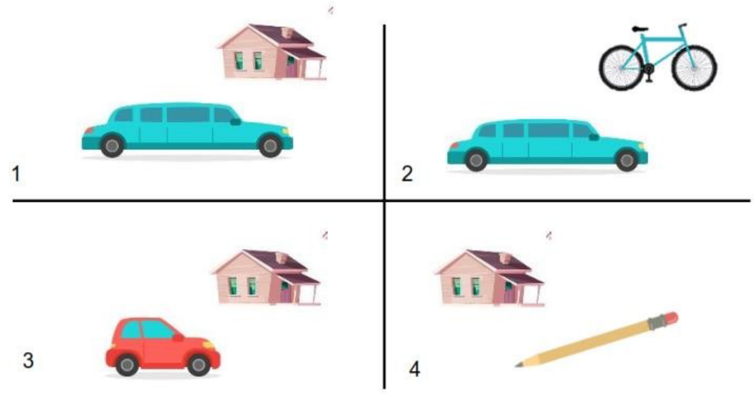<br>-	SI	NO
	**Mi dici cosa c'è in queste immagini?**<br>(**sc**a**rp**e) (ve**st**ito) (**gr**a**nd**e)<br>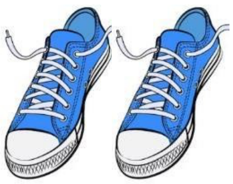 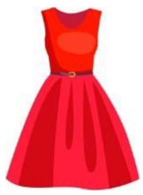 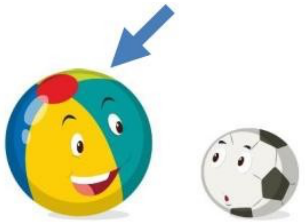<br>**Il bambino pronuncia bene tutte le lettere delle seguenti parole?***<br>*segnare NO se il bambino dice, ad esempio: "cappe", "scappe", "vettito, "vetito", "ande", "gande","gadde"…	SI	NO
	**Ripeti la frase dopo di me: "Il cane nero mangia un topo"**<br>*segnare NO se il bambino omette una o più parole	SI	NO

□ **IMPOSSIBILE COINVOLGERE IL BAMBINO**

TOTALE "NO": …./4

Progetto realizzato da Actis Maria e Bonzo Silvia (Logopediste - Università degli Studi di Torino)
In collaborazione con Dott.ssa E. Grosso (Logopedista), Dott.ssa G. De Cillis (Logopedista), Dott. A. Carpino (Pediatra)
