# Supplementary material for: Early Identification of DLD in Paediatric Practice: A Pilot Validation of the CLAP Screening Tool in Italian Outpatient Settings
Source: Int J Lang Commun Disord. 2026 Jun 25;61(4):e70282. doi: 10.1111/1460-6984.70282 (PMC13297869; doi:10.1111/1460-6984.70282)
Supplement: Supplementary file 3 — Supplementary Information: jlcd70282‐supp‐0003‐SuppMat.pdf [file JLCD-61-0-s002.pdf]

CLAP - Pediatrician

Communication and Language in the Pediatric Office Setting

Name and surname: Date of birth: Gender: □ M □ F

Identification number: Date of Completion:

24 – 30 months

□ Twin birth □ Bilingual/multilingual environment □ Attends kindergarten
First language(s):

Questions for PARENTS	**When the child speaks, is he/she easily understood by the family?**	YES	NO
	**Does the child say at least 15 everyday words (in any language he/she regularly uses)?***<br>For example: mommy, daddy, grandma, ball, woof woof, moo, house, etc.<br>*It doesn't matter how the child says them — mispronunciations (e.g., “wabbit” for “rabbit”) or onomatopoeic words (e.g., moo, vroom vroom) are acceptable.	YES	NO
	**Has the child started to say short phrases?**<br>For example: no food', 'give vroom vroom', 'not there'.<br><br>Mark NO if the child only speaks in single words such as 'food', 'give', or 'no'.	YES	NO
Questions for the CHILD	**Take… (the pen or the pencil or the car….)***<br><br>*Choose any object or toy available that is easily recognizable by the child.<br>Avoid using gestures that might help with understanding.<br><br>Mark NO if the child does not carry out the task or performs a different/incorrect one.	YES	NO
	**What’s in these pictures?**<br><br>(**m**um) *or* (**d**ad) *or* (**k**ids) (**d**og) (**c**at) (**m**ouse)<br>[1 word out of 3 is sufficient]<br><br>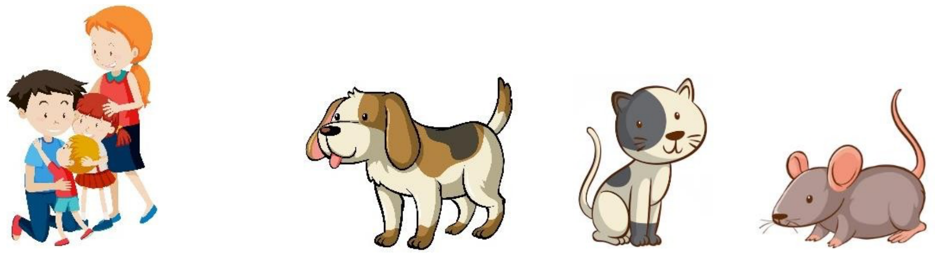<br><br>**Does the child pronounce correctly at least 3 of the letters in bold?**<br>Mark NO if the child does not respond or the sounds are not clearly pronounced.	YES	NO

□ **IMPOSSIBLE TO ENGAGE WITH THE CHILD**

TOTAL “NO”: …./5

CLAP - Pediatrician

Communication and Language in the Pediatric Office Setting

Name and surname:　　　　Date of birth:　　　　Gender: □ M □ F

Identification number:　　　　Date of Completion:

36 – 42 months

□ Twin birth　　　□ Bilingual/multilingual environment　　　□ Attends kindergarten

First language(s):

PARENTS	**When the child speaks, is he/she easily understood by people outside the family?***<br><br>* Meaning people who do NOT usually spend the day with him/her.	YES	NO
PEDIATRICIAN	**When the child speaks, do I easily understand him/her?**<br><br>Mark NO if the parents need to repeat or translate even just one word	YES	NO
Questions for the CHILD	**What's in these pictures?**<br><br>(**v**ase) *o* (**f**lower)　　(pa**c**i**f**ier)　　(i**c**e cream)　　(**s**un)<br>[1 word out of 2 is sufficient]<br><br>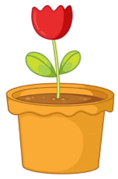 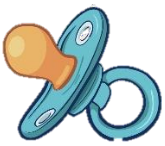 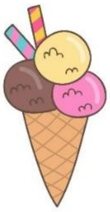 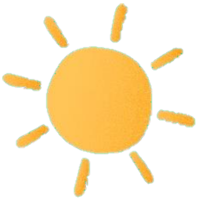<br><br>**Does the child pronounce correctly at least 3 of the letters in bold?**<br>Mark NO if the child mispronounce the sounds in bold, for example: "fase", "base", "power", "patipier", "ite", "tun" …	YES	NO
	**What are these children doing?**<br><br>The girl **sleeps**　　the boy **drinks** a coke　　the girl **washes** her hands<br><br>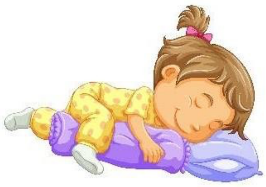 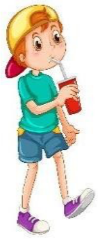 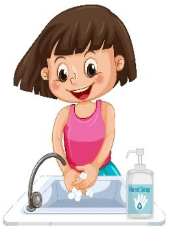<br><br>**Does the child describe what's in the pictures using verbs and/or combining multiple words?**<br>It doesn't matter *how* the child says them.	YES	NO

□ **IMPOSSIBLE TO ENGAGE WITH THE CHILD**

TOTAL "NO": …./4

CLAP - Pediatrician

Communication and Language in the Pediatric Office Setting

Name and surname: Date of birth: Gender: □ M □ F

Identification number: Date of Completion:

48 – 54 months

□ Twin birth □ Bilingual/multilingual environment □ Attends kindergarten

First language(s):

PARENTS	**When the child speaks, is he/she easily understood by people outside the family?***<br><br>* Meaning people who do NOT usually spend the day with him/her.	YES	NO
	**Has she/he started to say complete sentences (with articles, prepositions, etc.)?**<br>Example: "I want the ice cream I like so much"; "Dad takes Grandpa's bike".	YES	NO
PEDIATRICIAN	**When the child speaks, do I easily understand him/her?**<br><br>Mark NO if the parents need to repeat or translate even just one word	YES	NO
Questions for the CHILD	**Which is the correct picture?**<br><br>**"the cat is on the box"**<br>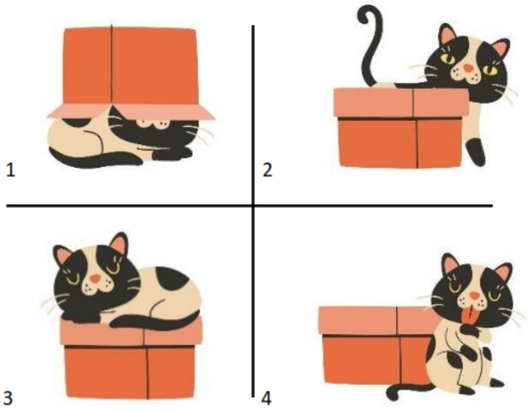 	YES	NO
	**What's in these pictures?**<br><br>(**st**ar) (pa**st**a) (**tr**ee) (mu**shr**ooms)<br>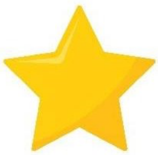 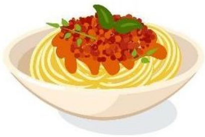 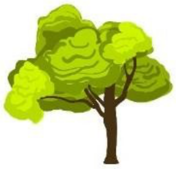 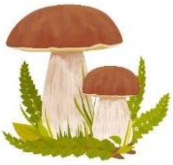<br><br>**Does the child pronounce at least 3 of the groups of letters in bold correctly?**<br><br>Mark NO if the child mispronounce the sounds in bold, for example: "tar", "patta", "passa", "tee", "mushoom", …	YES	NO

□ **IMPOSSIBLE TO ENGAGE WITH THE CHILD**

TOTAL "NO": …./5

CLAP - Pediatrician

Communication and Language in the Pediatric Office Setting

UNIVERSITÀ DI TORINO

Name and surname: Date of birth: Gender: □ M □ F

Identification number: Date of Completion:

60 – 72 months

□ Twin birth □ Bilingual/multilingual environment □ Attends kindergarten

First language(s):

PEDIATRICIAN	**Do I understand the child when she/he speaks to me?**<br><br>Mark NO if the parent has to repeat or translate even just one word	YES	NO
Questions for the CHILD	**Which is the correct picture?**<br><br>**“there is a long car and a small house”**<br>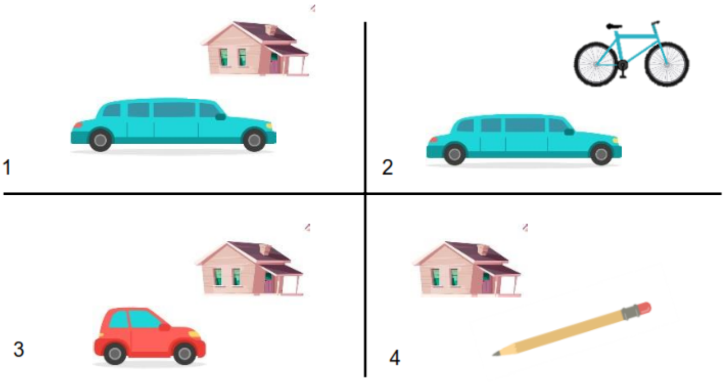	YES	NO
	**What’s in these pictures?**<br>(**sh**oes) (**dr**ess) (big)<br>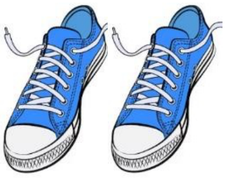 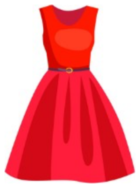 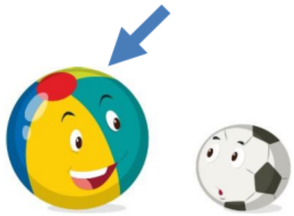<br>**Does the child pronounce all the letters of the following words correctly?***<br>*Mark NO if the child mispronounce the sounds in bold, for example: “sus”, “des”, “bik”…	YES	NO
	**Repeat the following sentence after me: “The black dog eats a mouse”**<br><br>*mark NO if the child omits one or more words	YES	NO

□ **IMPOSSIBLE TO ENGAGE WITH THE CHILD**

TOTAL “NO”: …./4
